# Association of Vitamin D Receptor Gene Polymorphisms with Serum 25-Hydroxyvitamin D Levels in Lithuanian Adults with Atopic Dermatitis: A Case—Control Study

**DOI:** 10.3390/ijms27104217

**Published:** 2026-05-09

**Authors:** Kamilija Briedė, Daina Pavalkienė, Brigita Gradauskienė, Agnė Bartnykaitė, Julius Leonavičius, Rasa Ugenskienė, Dalia Lukšienė, Vacis Tatarūnas, Skaidra Valiukevičienė

**Affiliations:** 1 Department of Skin and Venereal Diseases, Medical Academy, Lithuanian University of Health Sciences, LT-44307 Kaunas, Lithuania; 2 Department of Immunology and Allergology, Medical Academy, Lithuanian University of Health Sciences, LT-44307 Kaunas, Lithuania; 3 Lab of Immunology, Department of Immunology and Allergology, Lithuanian University of Health Sciences, LT-50161 Kaunas, Lithuania; 4 Oncology Research Laboratory, Oncology Institute, Lithuanian University of Health Sciences, LT-44307 Kaunas, Lithuania; 5 Department of Genetics and Molecular Medicine, Lithuanian University of Health Sciences, LT-44307 Kaunas, Lithuania; 6 Laboratory of Population Studies, Institute of Cardiology, Lithuanian University of Health Sciences, LT-50103 Kaunas, Lithuania; 7 Laboratory of Molecular Cardiology, Institute of Cardiology, Lithuanian University of Health Sciences, LT-50103 Kaunas, Lithuania

**Keywords:** atopic dermatitis, vitamin D receptor, *VDR*, single nucleotide polymorphisms, vitamin D status, eosinophils, immunoglobulin E

## Abstract

Atopic dermatitis (AD) is a chronic inflammatory skin disease increasingly prevalent in adults. Vitamin D plays an important role in regulating immune responses, cellular differentiation, and inflammation. Several single-nucleotide polymorphisms (SNPs) in the vitamin D receptor (*VDR*) gene have been suggested as biomarkers of AD susceptibility and severity. The aim of this study was to investigate six SNPs in the *VDR* gene (rs3847987, rs731236, rs7975232, rs1544410, rs2228570, and rs11168293) and their association with AD and blood biomarkers. Genotyping was performed in 91 adult patients with AD and 102 controls using real-time polymerase chain reaction. The genotype and allele distributions did not differ significantly between AD patients and controls. However, the G and T alleles of *VDR* rs731236 and rs1544410 were more frequently detected in individuals with serum 25-hydroxyvitamin D (25(OH)D) levels above 30 ng/mL. In contrast, the *VDR* rs7975232 C allele appears to be associated with lower odds of having a serum 25(OH)D level above 30 ng/mL. In genotype-stratified analysis, the T allele of *VDR* rs11168293 was more prevalent among individuals with eosinophil counts of 300 cells/μL. These findings suggest that *VDR* polymorphisms may contribute to variability in vitamin D status and inflammatory responses in adults with AD.

## 1. Introduction

Atopic dermatitis (AD) is a chronic inflammatory skin disease characterized by intense pruritus, eczematous lesions, and type 2 helper T lymphocyte (Th2)-dominant inflammation. Worldwide, up to 10% of adults are affected by AD [1]. In adults, AD is characterized by a more chronic disease course and is associated with a significant psychological burden [2,3].

Growing evidence suggests that serum 25-hydroxyvitamin D (25(OH)D) plays an important role in the pathophysiology of AD. A recent systematic review and meta-analysis showed that lower serum 25(OH)D levels are associated with greater AD severity, while vitamin D supplementation may improve clinical symptoms [4]. Suboptimal 25(OH)D3 levels are recognized as a modifiable risk factor for AD, with vitamin D3 supplementation demonstrating therapeutic potential in improving clinical outcomes [5]. In addition, several studies found that individuals with serum 25(OH)D insufficiency tend to have higher serum immunoglobulin E (IgE) levels and increased eosinophil count in blood compared to individuals with normal vitamin D levels [6,7].

The biological effects of serum 25(OH)D are primarily mediated through the vitamin D receptor (VDR), an intracellular nuclear receptor expressed in keratinocytes, immune cells, and various other tissues. After binding the active vitamin D metabolite 1,25-dihydroxyvitamin D_3_ (1,25(OH)_2_D_3_), VDR regulates multiple biological processes, including keratinocyte proliferation, epidermal differentiation, immune responses, and maintenance of skin barrier integrity [8,9,10]. These pleiotropic mechanisms are particularly relevant to the pathogenesis of chronic inflammatory skin diseases, including AD.

Genetic variation within the *VDR* gene may modulate vitamin D’s biological activity by influencing receptor function and activation. Given the important role of vitamin D in immune regulation and epidermal barrier homeostasis, *VDR* polymorphisms have attracted considerable attention as potential genetic factors contributing to inflammatory and atopy-associated disorders, including AD, asthma, and allergic rhinitis [11]. Multiple studies have demonstrated associations between *VDR* polymorphisms and the risk of atopic diseases, including AD, across populations from diverse geographic regions. Although several studies have investigated the association between *VDR* polymorphisms and AD, much of the available evidence derives from pediatric or mixed-age populations [11,12]. In contrast, studies conducted specifically in adult cohorts remain limited and have yielded inconsistent findings [13,14,15,16,17].

Several studies have demonstrated significant associations between *VDR* gene polymorphisms and atopic diseases, particularly involving variants such as rs2228570 and rs731236 [13,15]. Despite this, research focusing on these polymorphisms in adults remains scarce, and the findings on their association with atopic diseases are notably inconsistent. These discrepancies likely reflect substantial heterogeneity among study populations, including differences in age, sex, ethnic background, sample size, and clinical phenotype [18,19]. In addition to demographic and genetic factors, environmental and lifestyle influences also contribute to differences between study populations. Lithuania, as a high-latitude country in Northern Europe, receives limited ultraviolet B (UVB) radiation for much of the year, especially in autumn and winter, which reduces vitamin D synthesis in the skin [20,21]. The Baltic region experiences significant seasonal variation in sunlight, causing fluctuations in serum 25(OH)D levels throughout the year [16]. Additionally, dietary intake of vitamin D-rich foods, such as fatty fish and fortified products, is relatively low in Northern and Eastern Europe compared to Southern Europe [17]. These factors may lead to a higher prevalence of vitamin D insufficiency and help explain population-specific differences in vitamin D status and its interaction with *VDR* genetic variability, especially when compared to countries like Italy or Turkey, where sun exposure and dietary habits differ. Associations between *VDR* polymorphisms and AD may partly reflect gene–environment interactions rather than direct genetic effects alone [16,17].

Thus far, only a limited number of studies have examined associations between *VDR* polymorphisms and AD in adults, including investigations conducted in Italy, Turkey, Germany, and Lithuania [13,14,15,16,17]. Expanding research in this field may improve our understanding of the genetic factors underlying AD and may contribute to the development of more personalized prevention and treatment in adults with AD. Genetic variation in the *VDR* gene may influence vitamin D signaling by altering receptor expression, messenger RNA (mRNA) stability, or transcriptional activity. Several *VDR* polymorphisms have therefore been investigated in immune-mediated and allergic diseases, including AD [9,11,19]. In the present study, six *VDR* variants were selected based on previous evidence, biological plausibility, and relevance to European populations. Four canonical variants—rs731236, rs7975232, rs1544410, and rs2228570—have been studied in relation to AD susceptibility, disease severity, and vitamin D metabolism [12,13,15]. Among these, rs2228570 is a functional polymorphism located at the translation initiation site and may alter VDR protein structure and downstream receptor activity [19], whereas rs731236, rs7975232, and rs1544410, located in the 3′ region of the gene, may influence mRNA stability, gene expression, or linkage with other regulatory variants [11,12]. In addition, rs3847987 and rs11168293 were included because of reported associations with vitamin D status and inflammatory biomarkers in atopy-related phenotypes [14,16]. Thus, the selected SNP panel enabled comparison with previous studies and exploration of additional *VDR* genetic variability in an adult Baltic population.

Therefore, the present study was designed to assess associations of six *VDR* gene polymorphisms (rs3847987, rs731236, rs7975232, rs1544410, rs2228570, and rs11168293) with AD and AD-linked peripheral blood markers in a homogeneous adult population. We hypothesized that selected *VDR* polymorphisms may be associated with variability in serum 25(OH)D levels and AD-related peripheral blood biomarkers in Lithuanian adults.

## 2. Results

### 2.1. Clinical Characterization of Participants

The demographic data of the participants are summarized in Table 1. Cases and controls did not vary substantially by gender (*p* = 0.498) and age (*p* = 0.068) (Table 1). The main baseline clinical characteristics of the AD subjects are presented in Table 2.

Among the 91 patients with AD, 51 (56.0%) had serum 25(OH)D levels less than 30 ng/mL, 45 (49.5%) had elevated total IgE levels (>100 kU/L), and 36 (39.6%) had eosinophil counts above 300 cells/μL. The median values of serum 25(OH)D level, total IgE level, and eosinophil count in men and women are presented in Table 3. Men tended to have higher values of all analytes than women; however, the differences were not significant (*p* > 0.05) (Table 3).

In the analyzed cohort, 26 patients (28.6%) had the highest SCORAD index (>50). This group demonstrated statistically significantly higher total IgE levels and eosinophil counts compared with the other two groups (*p* < 0.001) (Table 3). Although eosinophil counts differed according to disease severity, genotype-based analysis additionally revealed an association between *VDR* rs11168293 and eosinophilia.

### 2.2. VDR Polymorphisms and AD

Six *VDR* gene polymorphisms (rs3847987, rs731236, rs7975232, rs1544410, rs2228570, and rs11168293) were investigated in both AD patients and control subjects. The distribution of all analyzed SNPs was consistent with the Hardy–Weinberg equilibrium (HWE) (*p* > 0.05) and is shown in Table 4.

The allele frequencies in the AD and control groups were comparable to those reported for European populations in the 1000 Genomes project. No significant differences in genotype or allele frequencies were observed between AD patients and controls (*p* > 0.05), indicating no association between the analyzed *VDR* polymorphisms and AD (Table 4).

### 2.3. VDR Polymorphisms and Serum 25(OH)D Levels

In the AD group, the serum 25(OH)D levels ranged from 3.87 to 107.46 ng/mL, with a median value of 27.42 ng/mL. Both quantitative and qualitative statistical analyses were conducted to compare the serum 25(OH)D levels across the *VDR* genotype and allele groups. The quantitative analysis showed no statistically significant differences for any of the analyzed SNPs (*p* > 0.05).

A serum 25(OH)D level of 30 ng/mL is typically regarded as the threshold for sufficiency [22,23,24,25,26,27], with lower levels associated with increased severity and a higher risk of AD. Consequently, for the qualitative analysis, AD patients were classified into two groups according to serum 25(OH)D-level differences: ≤30 and >30 ng/mL. This analysis demonstrated that certain *VDR* gene polymorphisms (rs731236, rs7975232, and rs1544410) were significantly associated with variations in serum 25(OH)D level (Table 5). AD patients carrying the rs731236 G allele had 1.867-fold higher odds of having a serum 25(OH)D level higher than 30 ng/mL (OR = 1.867, 95% CI: 1.011–3.448, *p* = 0.046). Similarly, univariate logistic regression analysis revealed that increased odds of serum 25(OH)D level > 30 ng/mL were significantly associated with the rs1544410 T allele (OR = 1.964, 95% CI: 1.064–3.624, *p* = 0.031). In contrast, in the analyzed AD patient group, the rs7975232 C allele was associated with decreased odds for vitamin D level > 30 ng/mL (OR = 0.523, 95% CI: 0.289–0.946, *p* = 0.032).

### 2.4. VDR Polymorphisms, Blood Eosinophils and Total IgE

Since the study showed significant differences in eosinophil count and total IgE levels among patients with different AD severities, further analyses were performed to investigate the association of *VDR* polymorphisms on both analytes. Total eosinophil counts and IgE levels were compared using quantitative and qualitative statistical analyses across different *VDR* genotype and allele groups.

As blood eosinophil counts greater than 300 cells/μL [28,29,30] and total IgE levels above 100 kU/L [31,32] are commonly considered elevated in clinical practice, these values were used as threshold levels for group stratification in the qualitative analysis. The results showed a significant association between the rs11168293 T allele and increased eosinophil count (*p* = 0.039, Table 6). In the qualitative analysis, it was confirmed that the rs11168293 T allele is associated with approximately 2-fold increased odds for eosinophil counts > 300 cells/μL (OR = 1.867, 95% CI: 1.006–3.464, *p* = 0.048) (Table 7). Analysis of the relationship between *VDR* gene SNPs and IgE levels showed that the heterozygous rs1544410 genotype was associated with lower odds of elevated IgE levels (>100 kU/L) (OR = 0.391, 95% CI: 0.157–0.975, *p* = 0.044) (Table 8).

## 3. Discussion

In recent years, increasing attention has been directed toward the role of serum 25(OH)D levels and vitamin D receptor (VDR) signaling in the pathophysiology of AD [5,9]. However, the associations between *VDR* gene polymorphisms and AD, as well as its clinical manifestations, remain unclear. Thus, in the present study, we investigated the association between six selected *VDR* gene polymorphisms (rs3847987, rs731236, rs7975232, rs1544410, rs2228570, rs11168293) and AD in a cohort of Lithuanian adults, as well as their relationship with serum 25(OH)D levels and markers of allergic inflammation. Although the analyzed polymorphisms were not associated with AD, several associations with blood biomarkers were observed. The threshold for vitamin D sufficiency is still debated in international guidelines. While some organizations, including the Institute of Medicine, recommend lower cut-offs, a serum 25(OH)D concentration of 30 ng/mL is widely used in clinical research and is commonly adopted in Endocrine Society guidance [24,25,26,27]. We selected this threshold to align with previous studies and because it may be clinically relevant for northern European populations, such as those in Lithuania, where seasonal ultraviolet exposure is limited. Specifically, our study showed that the *VDR* alternative alleles at rs731236 and rs1544410 were more frequently observed among patients with sufficient serum 25(OH)D levels (greater than 30 ng/mL). In contrast, there seems to be lower odds of having a serum 25(OH)D level more than 30 ng/mL when the *VDR* rs7975232 alternative allele is present. These results indicate that *VDR* genetic variation may contribute to differences in circulating 25(OH)D levels among patients with AD.

The observed association between certain alleles and higher serum 25(OH)D concentrations should not be viewed as direct protection against AD. Rather, AD is a multifactorial disorder influenced by epidermal barrier dysfunction, immune dysregulation, and environmental factors [1,8,9]. These variants may affect vitamin D metabolism or signaling pathways but are unlikely to be primary determinants of disease risk [11,19]. Such associations may also reflect compensatory biological mechanisms that modulate chronic Th2-skewed inflammation [9,19].

Furthermore, we observed a higher prevalence of the alternative allele of *VDR* rs11168293 among patients with an eosinophil count greater than 300 cells/μL, suggesting a potential link between this SNP and systemic allergic inflammation. Higher eosinophil counts and total IgE levels are well-established biomarkers of AD severity and reflect systemic immune activation [1,3,29]. This may indicate that *VDR* rs11168293 is involved in the modulation of type 2 immune responses. However, several studies have been contradictory and did not show a clear relationship between serum 25(OH)D levels and disease severity [6,7,14]. This discrepancy may be attributable to the relatively small sample size of the investigated subjects, limited statistical power, population-specific genetic and environmental factors, or differences in study design and methodologies of AD severity assessment.

To date, the selected *VDR* polymorphisms have been investigated in relatively few studies of atopic diseases, and the evidence remains inconsistent. For *VDR* rs3847987, a previous study similarly found no association with AD or allergic asthma, although genotype-specific differences in vitamin D status were reported [17]. Additionally, no significant relationship between *VDR* rs3847987 and several key inflammatory cytokines (IL-5, IL-17A, TGF-β1, IL-10, IFN-γ, IL-35, and IL-33) has been demonstrated [16]. These findings suggest that *VDR* rs3847987 may have a limited role in disease susceptibility but could still be involved in modulating pathways related to vitamin D.

Regarding *VDR* rs731236, our results align with those of other authors who observed a significant association between *VDR* rs731236 and serum 25(OH)D levels [14,15]. However, the immunological and clinical significance of *VDR* rs731236 remains uncertain. The associations between *VDR* rs731236 and levels of Th2 and Th17 cells, the Th1/Th2 ratio, serum IL-10 and TGF-β1 levels have been reported [16], while other studies found no relationship with AD or its clinical characteristics (disease onset, sex, IgE levels, generalized AD localization, etc.) [33,34]. This inconsistency may indicate that *VDR* rs731236 primarily influences regulation rather than clinical phenotype expression.

For *VDR* rs7975232, most previous studies have not determined an association with AD or its clinical features [14,34,35], although one study [33] reported a higher frequency of the *VDR* rs7975232 AA genotype in patients with earlier disease onset. Our findings extend these observations by suggesting a potential relationship with vitamin D insufficiency. This may indicate that *VDR* rs7975232 influences vitamin D metabolism or signaling rather than directly contributing to disease occurrence.

The evidence regarding *VDR* rs1544410 is also inconsistent. While studies in Chinese and Spanish populations did not demonstrate an association with AD susceptibility [34,35], a study in a Turkish population [15] showed a markedly increased risk in carriers of the *VDR* rs1544410 alternative genotype. In contrast, other studies found no association with clinical characteristics of AD [14,33]. Our results suggest that this SNP may have a role in serum 25(OH)D levels rather than disease susceptibility. *VDR* rs2228570 is a functional polymorphism located at the translation initiation site, resulting in an alternative VDR protein isoform that may modify receptor activity and downstream immune signaling [19]. Some studies have reported associations with AD susceptibility and IgE levels [34], but others did not observe significant relationships with disease occurrence or clinical features [15,33]. These discrepancies may reflect population-specific genetic backgrounds, environmental effects, and differences in study design. In our study, no significant association was identified, suggesting that rs2228570 may have a modest or situation-dependent role in AD rather than a direct effect on disease susceptibility or clinical manifestation.

Lastly, *VDR* rs11168293 has been linked to immune regulation in previous studies, where this SNP was associated with higher levels of IL-10 [16], eosinophil counts, and total IgE level [14]. Our findings of an association with eosinophilia are consistent with these observations and further support the role of the *VDR* rs11168293 polymorphism in modulating allergic inflammation. This may be explained by the role of *VDR* signaling in type 2 immune responses, where genetic variation could influence cytokine expression and eosinophil activation.

Among the studied variants, rs2228570 is a functional polymorphism. In contrast, rs1544410, rs7975232, and rs731236 are in the 3′ region of the *VDR* gene, suggesting that their effects may be mediated through regulatory mechanisms influencing mRNA stability or *VDR* expression levels. Similarly, rs3847987 and rs11168293 are intronic variants that are unlikely to directly affect protein structure but may act as regulatory polymorphisms or proxy markers for functional elements involved in transcriptional regulation, chromatin accessibility, or enhancer activity [36,37,38,39]. Through these mechanisms, genetic variation in *VDR* may modulate receptor expression, availability, and transcriptional efficiency, thereby influencing downstream target gene expression, including CYP24A1, a key enzyme involved in vitamin D catabolism, thereby contributing to interindividual differences in vitamin D metabolism and homeostasis [40,41].

A principal strength of this study is the comprehensive evaluation of multiple *VDR* polymorphisms in relation to both vitamin D status and clinically relevant inflammatory biomarkers within a well-defined adult AD population. In contrast to previous studies conducted in broader atopic cohorts [16,18], this investigation specifically addresses AD as a distinct clinical entity, thereby providing more targeted insight into disease-related mechanisms. Furthermore, our findings extend previous research by demonstrating that *VDR* polymorphisms may influence biological pathways relevant to AD, even in the absence of a direct association with disease occurrence. Several limitations should be acknowledged. First, the relatively small sample size may have limited the statistical power to detect associations between the investigated *VDR* polymorphisms and AD. Given the available sample size, the study had sufficient power to detect moderate-to-large effect sizes, but smaller genetic effects may have gone undetected. As a result, the lack of significant associations with AD susceptibility should be interpreted cautiously, especially for complex polygenic traits like AD. Achieving adequate statistical power (>80%) would likely require larger effect sizes (OR ≥ 1.7–2.0), which are uncommon in complex diseases. Second, only six polymorphisms within the *VDR* gene were analyzed, while other variants involved in vitamin D metabolism, including those related to synthesis, transport, and degradation, were not assessed and may also influence serum 25(OH)D levels and immune responses. We performed several statistical comparisons, which could have raised the chance of type I error. To address this, we used the Benjamini–Hochberg method for false-discovery rate (FDR) correction as a sensitivity analysis. Since this was an exploratory study focused on identifying possible genetic associations rather than providing final proof, we examined both nominal and FDR-adjusted *p*-values. After FDR correction, none of the associations stayed statistically significant. Because our sample size was small and the genetic models were related, the FDR method might have been overly strict, increasing the risk of type II error. For these reasons, our findings should be seen as exploratory, and we report FDR-adjusted results to be transparent and thorough. Third, environmental factors that may influence serum 25(OH)D concentrations, including dietary intake and sunlight exposure, were not controlled in the present study. Seasonal variation in sunlight exposure during the blood collection period was not accounted for, which may have contributed to variability in vitamin D levels independently of *VDR* genetic variation. This is particularly relevant in high-latitude regions such as Lithuania, where sunlight exposure changes markedly across seasonal fluctuations in UV radiation which may substantially limit cutaneous vitamin D synthesis during much of the year. Furthermore, local dietary patterns and variability in the consumption of vitamin D-rich or fortified foods may also affect serum 25(OH)D concentrations [21,22]. These regional factors may modify the association between *VDR* polymorphisms and vitamin D status in this cohort. Additionally, key Th2 cytokines, including IL-4, IL-5, and IL-13, were not evaluated in this study. Future investigations incorporating cytokine profiling may provide a more comprehensive understanding of the relationship between *VDR* polymorphisms and immune dysregulation in AD. Despite these limitations, this study provides new insights into the relationship between *VDR* polymorphisms, serum 25(OH)D status, and biomarkers in adults with AD from the Baltic region, where genetic studies on this topic remain limited.

This study did not identify a significant association between the investigated *VDR* polymorphisms and AD susceptibility in Lithuanian adults. However, several variants showed significant relationships with biological markers relevant to disease activity: rs731236 and rs1544410 were associated with higher odds of sufficient serum 25(OH)D levels, rs7975232 with lower odds of vitamin D sufficiency, and rs11168293 with eosinophilia. These results suggest that *VDR* genetic variation may act more as a modifier of disease-related biomarkers and phenotype expression than as a primary driver of AD susceptibility. Clinically, these variants may account for interindividual differences in vitamin D status and immune responses among adults with AD. Further multicenter studies with adequate statistical power are required to confirm these data.

## 4. Materials and Methods

### 4.1. Study Population

A total of 193 participants (91 patients with AD and 102 controls) were enrolled in this case–control study. Patients were recruited at the Department of Skin and Venereal Diseases, Hospital of the Lithuanian University of Health Sciences Kauno Klinikos, from 2022 to 2024. Diagnosis of AD was confirmed according to the Hanifin and Rajka criteria by one dermatovenereologist (K.B.). The study was approved by the Kaunas Regional Biomedical Research Ethics Committee (No. BE-2-74). A blank informed consent form is provided in the Supplementary Materials. The inclusion criteria for the case group were a confirmed diagnosis of AD, age 18–60 years, no use of systemic immunosuppressive medications for at least 1 month prior to study enrolment, no use of vitamin D supplements for at least three months prior to enrolment, and absence of malignant disease, autoimmune disorders, chronic or acute infections. Exclusion criteria were age <18 years or >60 years, use of systemic immunosuppressive medications within 1 month prior to examination, presence of malignancy, autoimmune disease, chronic or acute infections, participation in other clinical trials and refusal or inability to provide informed consent due to psychological or other reasons. The control group included individuals without a history of AD. In addition, participants completed a structured questionnaire on chronic diseases, including questions about physician-diagnosed allergic conditions such as asthma, allergic rhinitis, and other atopy-related disorders. They were selected from respondents (*N* = 3426) who participated in the epidemiological health survey entitled “Chronic Diseases and Their Risk Factors in the Adult Population,” conducted in Kaunas, Lithuania. Eligibility criteria included age of 25 years or older, willingness to participate in the biomedical study, and absence of AD as confirmed by dermatological examination. Exclusion criteria applied to both case and control groups. These included severe systemic diseases, mental disorders that impaired understanding or participation, pregnancy, or lactation. Individuals who declined participation or withdrew consent during the study were also excluded.

### 4.2. Clinical Examination

All participants underwent an objective examination, including anthropometric measurements [42] and skin examination using both qualitative criteria of AD according to Hanifin and Rajka [43]. For subjects in the case group, the Scoring Atopic Dermatitis (SCORAD) index was used to assess AD severity and severity categories (mild, moderate, severe) were defined according to established interpretation guidelines, with cut-offs of <25, 25–50, and >50, respectively [44]. All SCORAD assessments were performed by the same dermatovenereolologist (the co-author K.B.) throughout the study to ensure consistency.

### 4.3. Blood Sampling and Sample Processing

Peripheral venous blood samples were obtained from all participants under standard conditions. Blood samples intended for genetic analysis and eosinophil count determination were collected in tubes containing the anticoagulant ethylenediaminetetraacetic acid (K3-EDTA). Samples for biochemical analyses, including serum 25-hydroxyvitamin D and total IgE concentrations, were collected in serum separation tubes (SSTs). Each specimen was assigned a unique identification code to ensure anonymization and accurate linkage between biological samples and clinical data. Serum tubes were centrifuged at 3500 rpm for 10 min, after which the serum fraction was separated and stored at −80 °C until further laboratory analysis.

### 4.4. Laboratory Measurements

Serum concentrations of vitamin D (25-hydroxyvitamin D, 25(OH)D) were determined using a commercially available enzyme-linked immunosorbent assay (ELISA) kit with a commercially available kit (BioVendor, Brno, Czech Republic). The inter-assay coefficient of variation (CV) for the assay was approximately 20%, and the analytical detection limit was 2.81 ng/mL. Vitamin D status was categorized as deficiency (<20 ng/mL), insufficiency (20–30 ng/mL), sufficient levels (30–50 ng/mL), and high levels (>50 ng/mL), according to commonly used clinical thresholds.

Peripheral blood eosinophil counts were measured using an automated hematology analyzer (Sysmex, Kobe, Japan). Serum total IgE concentrations were quantified using a commercially available ELISA kit (IBL International, Hamburg, Germany). The inter-assay coefficient of variation was 4.1%, and the assay detection limit was 0.8 IU/mL. Based on the manufacturer’s recommendations, total IgE levels below 100 IU/mL were considered within the normal range for adults.

### 4.5. DNA Extraction and SNP Genotyping

Genomic DNA extraction was performed using a QIAamp DNA blood mini kit (Qiagen, Hilden, Germany; Cat#51104). DNA concentration and purity were analyzed by Multiskan SkyHigh Microplate Spectrophotometer (Thermo Scientific, Waltham, MA, USA; Cat#A51119600DPC) and stored at −20 °C. Six SNPs (rs3847987, rs731236, rs7975232, rs1544410, rs2228570, and rs11168293) in the *VDR* gene were genotyped by using the QuantStudio 3 Real-Time PCR System (Applied Biosystems, Foster City, CA, USA; Cat#A28137). Table 9 lists the TaqMan SNP genotyping assay IDs used for the analysis. The provided genotyping assays were combined with TaqMan^®^ Universal Master Mix II with UNG (Applied Biosystems, Foster City, CA, USA), nuclease-free water, and genomic DNA. The reaction mixtures were incubated with a pre-read step at 60 °C for 30 s, followed by an initial denaturation at 95 °C for 10 min. This was followed by 40 amplification cycles consisting of 95 °C for 15 s and 60 °C for 1 min, with a post-read step at 60 °C for 30 s.

### 4.6. Statistical Analysis

Statistical Package for Social Sciences (IBM SPSS Statistics 30.0.0.0) software was used for statistical data analysis. Data normality was assessed using the Kolmogorov–Smirnov and Shapiro–Wilk tests depending on group size. Non-parametric data are presented as medians together with minimum–maximum values. The Mann–Whitney U and Kruskal–Wallis tests were used for comparing the data between groups. The assessment of Hardy–Weinberg equilibrium (HWE) was performed by a goodness-of-fit Chi-square test. Categorical variables were compared using the Chi-square test or Fisher’s exact test when expected cell counts were small. Logistic regression analyses were conducted to determine associations between analyzed polymorphisms in the *VDR* gene and AD. Moreover, association analyses were performed to assess the relationship between *VDR* gene polymorphisms and serum 25(OH)D-level groups, total IgE levels, and blood eosinophil counts. Correction for multiple testing was performed separately for each analyzed outcome (AD susceptibility, serum 25(OH)D level, IgE level, and eosinophil count) to control for the number of tests performed within each set of related hypotheses. For each outcome, *p*-values obtained from logistic regression analyses across all tested SNPs and genetic models were adjusted using the Benjamini–Hochberg false-discovery rate (FDR) method. The *p*-values, odds ratios (ORs), and 95% confidence intervals (CIs) were reported to quantify the strength of the associations.

## Figures and Tables

**Table 1:** General demographic characteristics of study participants with atopic dermatitis (n = 91) and control (n = 102) groups.

Characteristics	Number, n (%)	Gender			Age	
		Male, n (%)	Female, n (%)	*p*-Value	Years, Median (Min–Max)	*p*-Value
Controls	102 (100.0)	36 (35.3)	66 (64.7)	0.498	28 (23–45)	0.068
AD patients	91 (100.0)	27 (29.7)	64 (70.3)		27 (18–59)	

AD—atopic dermatitis.

**Table 2:** Clinical profile of the atopic dermatitis (AD) group (n = 91).

Variables	Distribution, n (%)
**AD symptom onset, n (%)**	
Early onset (0 to <5 years old)	48 (52.7)
Childhood (5 to <18 years old)	21 (23.1)
Adult (18 years old)	22 (24.2)
**Asthma, n (%)**	
Present	20 (22.0)
Absent	71 (78.0)
**Rhinitis, n (%)**	
Present	22 (24.2)
Absent	69 (75.8)
**Conjunctivitis, n (%)**	
Present	19 (20.9)
Absent	72 (79.1)
**Severity of AD by SCORAD index, n (%)**	
Mild (<25)	32 (35.2)
Moderate (25–50)	33 (36.3)
Severe (>50)	26 (28.6)
**Dermatology life quality index (DLQI), median (min–max)**	8 (0–28)
**Body mass index (BMI) (kg/m^2^), n (%)**	
Underweight (≤18.5)	5 (5.5)
Normal weight (18.5–24.9)	58 (63.7)
Overweight (25–29.9)	18 (19.8)
Obesity (≥30)	10 (11.0)
**Total IgE (kU/L), median (min–max)**	90.17 (3.00–13,739.50)
**Blood eosinophils count (% of leukocytes), median (min–max)**	3.80 (0.00–37.90)
**Blood eosinophils count (x10^9^/L), median (min–max)**	0.20 (0.00–3.00)
**Serum 25(OH)D level (ng/mL), median (min–max)**	27.42 (3.87–107.46)
Deficiency (<20 ng/mL)	21 (23.1)
Insufficiency (20–30 ng/mL)	30 (33.0)
Normal (30–50 ng/mL)	29 (31.9)
High (>50 ng/mL)	11 (12.1)

AD—atopic dermatitis; SCORAD—Scoring Atopic Dermatitis; IgE—immunoglobulin E; 25(OH)D—25-hydroxyvitamin D.

**Table 3:** Serum 25(OH)D levels, total IgE levels, and blood eosinophils in patients with different courses of AD.

Characteristics	Serum 25(OH)D Level (ng/mL)	*p*-Value	Total IgE Level (kU/L)	*p*-Value	Eosinophils Count (Cells/μL)	*p*-Value
Male (n = 27)	25.10 (3.87–107.46)	0.126	127.48 (3.50–13,739.50)	0.089	300 (100–3000)	0.763
Female (n = 64)	29.18 (6.01–81.33)		73.58 (3.00–9763.30)		200 (0–1400)	
SCORAD—Mild (<25) (n = 32)	27.95 (6.01–81.33)	0.978	38.90 (3.00–819.00)	**<0.001**	150 (100–1000)	**<0.001**
SCORAD—Moderate (25–50) (n = 33)	28.63 (11.75–64.20)		60.10 (3.00–6766.50)		200 (0–800)	
SCORAD—Severe (>50) (n = 26)	27.32 (3.87–107.46)		1064.80 (18.79–13,739.50)		600 (100–3000)	

25(OH)D—25-hydroxyvitamin D; IgE—immunoglobulin E; SCORAD—Scoring Atopic Dermatitis. Results are presented as median (minimum–maximum) values. Bold represents statistically significant values.

**Table 4:** Genotype analysis of *VDR* gene polymorphisms in AD patients (n = 91) and controls (n = 102).

SNP	Genotype/Allele	AD Patients, n (%)	Controls, n (%)	*p*-Value	OR (95% CI)	*p*-Value	Adjusted *p*-Value
rs3847987	CC	72 (79.1)	83 (81.4)	0.781	1.000		
	CA	19 (20.9)	18 (17.6)		1.217 (0.594–2.494)	0.592	0.874
	AA	0 (0.0)	1 (1.0)		N/A		
	CA + AA	19 (20.9)	19 (18.6)	0.833	1.153 (0.567–2.345)	0.695	0.874
	C	163 (89.6)	184 (90.2)	0.890	1.000		
	A	19 (10.4)	20 (9.8)		1.072 (0.553–2.080)	0.836	
rs731236	AA	38 (41.8)	40 (39.2)	0.789	1.000		
	AG	41 (45.1)	45 (44.1)		0.959 (0.519–1.771)	0.894	0.894
	GG	12 (13.2)	17 (16.7)		0.743 (0.314–1.760)	0.500	0.874
	AG + GG	53 (58.2)	62 (60.8)	0.832	0.900 (0.506–1.600)	0.719	0.874
	A	117 (64.3)	125 (61.3)	0.906	1.000		
	G	65 (35.7)	79 (38.7)		0.879 (0.581–1.330)	0.542	0.874
rs7975232	AA	20 (22.0)	26 (25.5)	0.842	1.000		
	AC	46 (50.5)	50 (49.0)		1.196 (0.590–2.426)	0.620	0.874
	CC	25 (27.5)	26 (25.5)		1.250 (0.561–2.784)	0.585	0.874
	AC + CC	71 (78.0)	76 (74.5)	0.687	1.214 (0.624–2.366)	0.568	0.874
	A	86 (47.3)	102 (50.0)	0.903	1.000		
	C	96 (52.7)	102 (50.0)		1.116 (0.748–1.665)	0.590	0.874
rs1544410	CC	38 (41.8)	39 (38.2)	0.783	1.000		
	CT	40 (44.0)	45 (44.1)		0.912 (0.492–1.691)	0.771	0.874
	TT	13 (14.3)	18 (17.6)		0.741 (0.319–1.720)	0.486	0.874
	CT + TT	53 (58.2)	63 (61.8)	0.725	0.863 (0.485–1.538)	0.618	0.874
	C	116 (63.7)	123 (60.3)	0.827	1.000		
	T	66 (36.3)	81 (39.7)		0.864 (0.572–1.305)	0.487	0.874
rs2228570	GG	27 (29.7)	34 (33.3)	0.819	1.000		
	GA	44 (48.4)	45 (44.1)		1.231 (0.640–2.368)	0.533	0.874
	AA	20 (22.0)	23 (22.5)		1.095 (0.500–2.398)	0.820	0.874
	GA + AA	64 (70.3)	68 (66.7)	0.696	1.185 (0.644–2.181)	0.585	0.874
	G	98 (53.8)	113 (55.4)	0.865	1.000		
	A	84 (46.2)	91 (44.6)		1.064 (0.712–1.590)	0.761	0.874
rs11168293	GG	39 (42.9)	32 (31.4)	0.255	1.000		
	GT	39 (42.9)	52 (51.0)		0.615 (0.329–1.150)	0.128	0.874
	TT	13 (14.3)	18 (17.6)		0.593 (0.253–1.390)	0.229	0.874
	GT + TT	52 (57.1)	70 (68.6)	0.133	0.610 (0.338–1.099)	0.100	0.874
	G	117 (64.3)	116 (56.9)	0.208	1.000		
	T	65 (35.7)	88 (43.1)		0.732 (0.486–1.104)	0.137	0.874

SNP—single-nucleotide polymorphism; AD—atopic dermatitis; OR—odds ratio; CI—confidence interval; N/A—not available; adjusted *p*-value refers to *p*-values corrected for multiple comparisons using the Benjamini–Hochberg false-discovery rate (FDR) approach.

**Table 5:** Association between serum 25(OH)D-level groups (≤30 and >30 ng/mL) and *VDR* gene polymorphisms.

SNP	Genotype/Allele	≤30 ng/mL, n (%)	>30 ng/mL, n (%)	*p*-Value	OR (95% CI)	*p*-Value	Adjusted *p*-Value
rs3847987	CC	41 (80.4)	31 (77.5)	0.736	1.000		
	CA	10 (19.6)	9 (22.5)		1.190 (0.432–3.282)	0.736	0.824
	AA	0 (0.0)	0 (0.0)		N/A		
	CA + AA	10 (19.6)	9 (22.5)	0.939	1.190 (0.432–3.282)	0.736	0.824
	C	92 (90.2)	71 (88.8)	0.942	1.000		
	A	10 (9.8)	9 (11.3)		1.166 (0.450–3.022)	0.752	0.824
rs731236	AA	25 (49.0)	13 (32.5)	0.131	1.000		
	AG	22 (43.1)	19 (47.5)		1.661 (0.669–4.121)	0.274	0.473
	GG	4 (7.8)	8 (20.0)		3.846 (0.973–15.207)	0.055	0.181
	AG + GG	26 (51.0)	27 (67.5)	0.170	1.997 (0.845–4.718)	0.115	0.240
	A	72 (70.6)	45 (56.3)	0.065	1.000		
	G	30 (29.4)	35 (43.8)		1.867 (1.011–3.448)	**0.046**	0.176
rs7975232	AA	7 (13.7)	13 (32.5)	0.075	1.000		
	AC	27 (52.9)	19 (47.5)		0.379 (0.127–1.127)	0.081	0.233
	CC	17 (33.3)	8 (20.0)		0.253 (0.073–0.880)	**0.031**	0.166
	AC + CC	44 (86.3)	27 (67.5)	0.059	0.330 (0.117–0.931)	**0.036**	0.166
	A	41 (40.2)	45 (56.3)	**0.045**	1.000		
	C	61 (59.8)	35 (43.8)		0.523 (0.289–0.946)	**0.032**	0.166
rs1544410	CC	25 (49.0)	13 (32.5)	0.088	1.000		
	CT	22 (43.1)	18 (45.0)		1.573 (0.630–3.928)	0.332	0.509
	TT	4 (7.8)	9 (22.5)		4.327 (1.116–16.776)	**0.034**	0.166
	CT + TT	26 (51.0)	27 (67.5)	0.170	1.997 (0.845–4.718)	0.115	0.240
	C	72 (70.6)	44 (55.0)	**0.044**	1.000		
	T	30 (29.4)	36 (45.0)		1.964 (1.064–3.624)	**0.031**	0.166
rs2228570	GG	17 (33.3)	10 (25.0)	0.521	1.000		
	GA	22 (43.1)	22 (55.0)		1.700 (0.638–4.527)	0.288	0.473
	AA	12 (23.5)	8 (20.0)		1.133 (0.346–3.716)	0.836	0.873
	GA + AA	34 (66.7)	30 (75.0)	0.527	1.500 (0.596–3.774)	0.389	0.559
	G	56 (54.9)	42 (52.5)	0.863	1.000		
	A	46 (45.1)	38 (47.5)		1.101 (0.612–1.981)	0.747	0.824
rs11168293	GG	19 (37.3)	20 (50.0)	0.207	1.000		
	GT	25 (51.0)	14 (32.5)		0.475 (0.190–1.186)	0.111	0.240
	TT	6 (11.8)	7 (17.5)		1.108 (0.315–3.901)	0.873	0.873
	GT + TT	32 (62.7)	20 (50.0)	0.314	0.594 (0.256–1.376)	0.224	0.429
	G	64 (62.7)	53 (66.3)	0.738	1.000		
	T	38 (37.3)	27 (33.8)		0.858 (0.465–1.584)	0.624	0.824

SNP—single-nucleotide polymorphism; N/A—not available; OR—odds ratio; CI—confidence interval; bold represents statistically significant values; adjusted *p*-value refers to *p*-values corrected for multiple comparisons using the Benjamini–Hochberg false-discovery rate (FDR) approach.

**Table 6:** The distribution of eosinophil count (cells/μL) according to *VDR* gene polymorphism genotypes and alleles.

SNP	Homozygous (Ref.), Cells/μL	Heterozygous, Cells/μL	Homozygous (Alt.), Cells/μL	*p*-Value	Allele (Ref.), Cells/μL	Allele (Alt.), Cells/μL	*p*-Value
rs3847987	CC	CA	AA	0.335	C	A	0.390
	200 (0–1400)	200 (100–3000)	N/A		200 (0–3000)	200 (100–3000)	
rs731236	AA	AG	GG	0.931	A	G	0.644
	200 (0–1000)	300 (100–300)	200 (100–1400)		200 (0–3000)	200 (100–3000)	
rs7975232	AA	AC	CC	0.772	A	C	0.162
	200 (100–3000)	250 (100–1100)	200 (0–1000)		200 (100–3000)	200 (0–1100)	
rs1544410	CC	CT	TT	0.953	C	T	0.554
	200 (0–1000)	250 (100–3000)	200 (100–1400)		200 (0–3000)	200 (100–3000)	
rs2228570	GG	GA	AA	0.712	G	A	0.603
	300 (0–1100)	200 (100–3000)	200 (100–900)		200 (0–3000)	200 (100–3000)	
rs11168293	GG	GT	TT	0.105	G	T	**0.039**
	200 (100–1400)	200 (0–1000)	500 (100–3000)		200 (0–1400)	300 (0–3000)	

SNP—single-nucleotide polymorphism; ref.—reference; alt.—alternative. Results are presented as median (minimum–maximum) values. Bold represents statistically significant values.

**Table 7:** Association between eosinophil count groups (≤300 and >300 cells/μL) and *VDR* gene polymorphisms.

SNP	Genotype/Allele	≤300 Cells/μL	>300 Cells/μL	*p*-Value	OR (95% CI)	*p*-Value	Adjusted *p*-Value
rs3847987	CC	45 (81.8)	27 (75.0)	0.434	1.000		
	CA	10 (18.2)	9 (25.0)		1.500 (0.541–4.156)	0.436	0.995
	AA	0 (0.0)	0 (0.0)		N/A		
	CA + AA	10 (18.2)	9 (25.0)	0.604	1.500 (0.541–4.156)	0.436	0.995
	C	100 (90.9)	63 (87.5)	0.626	1.000		
	A	10 (9.1)	9 (12.5)		1.429 (0.550–3.709)	0.464	0.995
rs731236	AA	23 (41.8)	15 (41.7)	0.500	1.000		
	AG	23 (41.8)	18 (50.0)		1.200 (0.490–2.941)	0.690	0.995
	GG	9 (16.4)	3 (8.3)		0.511 (0.119–2.200)	0.367	0.995
	AG + GG	32 (58.2)	21 (58.3)	1.000	1.006 (0.429–2.359)	0.989	0.995
	A	69 (62.7)	48 (66.7)	0.701	1.000		
	G	41 (37.3)	24 (33.3)		0.841 (0.451–1.571)	0.588	0.995
rs7975232	AA	13 (23.6)	7 (19.4)	0.740	1.000		
	AC	26 (47.3)	20 (55.6)		1.429 (0.481–4.241)	0.521	0.995
	CC	16 (29.1)	9 (25.0)		1.045 (0.306–3.571)	0.944	0.995
	AC + CC	42 (76.4)	29 (80.6)	0.831	1.282 (0.456–3.605)	0.637	0.995
	A	52 (47.3)	34 (47.2)	1.000	1.000		
	C	58 (52.7)	38 (52.8)		1.002 (0.553–1.817)	0.995	0.995
rs1544410	CC	23 (41.8)	15 (41.7)	0.754	1.000		
	CT	23 (41.8)	17 (47.2)		1.133 (0.459–2.797)	0.786	0.995
	TT	9 (16.4)	4 (11.1)		0.681 (0.177–2.617)	0.576	0.995
	CT + TT	32 (58.2)	21 (58.3)	1.000	1.006 (0.429–2.359)	0.989	0.995
	C	69 (62.7)	47 (65.3)	0.848	1.000		
	T	41 (37.3)	25 (34.7)		0.895 (0.481–1.665)	0.726	0.995
rs2228570	GG	16 (29.1)	11 (30.6)	0.600	1.000		
	GA	25 (45.5)	19 (52.8)		1.105 (0.418–2.923)	0.840	0.995
	AA	14 (25.5)	6 (16.7)		0.623 (0.183–2.125)	0.450	0.995
	GA + AA	39 (70.9)	25 (69.4)	1.000	0.932 (0.373–2.333)	0.881	0.995
	G	57 (51.8)	41 (56.9)	0.599	1.000		
	A	53 (48.2)	31 (43.1)		0.813 (0.447–1.479)	0.498	0.995
rs11168293	GG	27 (49.1)	12 (33.3)	0.141	1.000		
	GT	23 (41.8)	16 (44.4)		1.565 (0.616–3.977)	0.346	0.995
	TT	5 (9.1)	8 (22.2)		3.600 (0.973–13.316)	0.055	0.633
	GT + TT	28 (50.9)	24 (66.7)	0.205	1.929 (0.807–4.611)	0.140	0.995
	G	77 (70.0)	40 (55.6)	0.067	1.000		
	T	33 (30.0)	32 (44.4)		1.867 (1.006–3.464)	**0.048**	0.633

SNP—single-nucleotide polymorphism; OR—odds ratio; CI—confidence interval; N/A—not available. Bold represents statistically significant values; adjusted *p*-value refers to *p*-values corrected for multiple comparisons using the Benjamini–Hochberg false-discovery rate (FDR) approach.

**Table 8:** Association between IgE level groups (≤100 and >100 kU/L) and *VDR* gene polymorphisms.

SNP	Genotype/Allele	≤100 kU/L	>100 kU/L	*p*-Value	OR (95% CI)	*p*-Value	Adjusted *p*-Value
rs3847987	CC	36 (78.3)	36 (80.0)	1.000	1.000		
	CA	10 (21.7)	9 (20.0)		0.900 (0.327–2.476)	0.838	0.848
	AA	0 (0.0)	0 (0.0)		N/A		
	CA + AA	10 (21.7)	9 (20.0)	1.000	0.900 (0.327–2.476)	0.838	0.848
	C	82 (89.1)	81 (90.0)	1.000	1.000		
	A	10 (10.9)	9 (10.0)		0.911 (0.352–2.359)	0.848	0.848
rs731236	AA	15 (32.6)	23 (51.1)	0.161	1.000		
	AG	25 (54.3)	16 (35.6)		0.417 (0.169–1.031)	0.058	0.288
	GG	6 (13.0)	6 (13.3)		0.652 (0.177–2.406)	0.521	0.725
	AG + GG	31 (67.4)	22 (48.9)	0.115	0.463 (0.198–1.082)	0.075	0.288
	A	55 (59.8)	62 (68.9)	0.260	1.000		
	G	37 (40.2)	28 (31.1)		0.671 (0.365–1.236)	0.201	0.514
rs7975232	AA	14 (30.4)	6 (13.3)	0.135	1.000		
	AC	20 (43.5)	26 (57.8)		3.033 (0.990–9.297)	0.052	0.288
	CC	12 (26.1)	13 (28.9)		2.528 (0.734–8.709)	0.142	0.467
	AC + CC	32 (69.6)	39 (86.7)	0.086	2.844 (0.981–8.245)	0.054	0.288
	A	48 (52.2)	38 (42.2)	0.232	1.000		
	C	44 (47.8)	52 (57.8)		1.493 (0.832–2.680)	0.180	0.514
rs1544410	CC	15 (32.6)	23 (51.1)	0.119	1.000		
	CT	25 (54.3)	15 (33.3)		0.391 (0.157–0.975)	**0.044**	0.288
	TT	6 (13.0)	7 (15.6)		0.761 (0.214–2.709)	0.673	0.815
	CT + TT	31 (67.4)	22 (48.9)	0.115	0.463 (0.198–1.082)	0.075	0.288
	C	55 (59.8)	61 (67.8)	0.333	1.000		
	T	37 (40.2)	29 (32.2)		0.707 (0.385–1.297)	0.263	0.583
rs2228570	GG	15 (32.6)	12 (26.7)	0.545	1.000		
	GA	23 (50.0)	21 (46.7)		1.141 (0.436–2.988)	0.788	0.848
	AA	8 (17.4)	12 (26.7)		1.875 (0.580–6.061)	0.294	0.583
	GA + AA	31 (67.4)	33 (73.3)	0.696	1.331 (0.539–3.285)	0.536	0.725
	G	53 (57.6)	45 (50.0)	0.378	1.000		
	A	39 (42.4)	45 (50.0)		1.359 (0.757–2.438)	0.304	0.583
rs11168293	GG	18 (39.1)	21 (46.7)	0.768	1.000		
	GT	21 (45.7)	18 (40.0)		0.735 (0.302–1.790)	0.497	0.725
	TT	7 (15.2)	6 (13.3)		0.735 (0.209–2.588)	0.631	0.806
	GT + TT	28 (60.9)	24 (53.3)	0.607	0.735 (0.319–1.690)	0.468	0.725
	G	57 (62.0)	60 (66.7)	0.611	1.000		
	T	35 (38.0)	30 (33.3)		0.814 (0.443–1.495)	0.508	0.725

SNP—single-nucleotide polymorphism; OR—odds ratio; CI—confidence interval; N/A—not available. Bold represents statistically significant values; adjusted *p*-value refers to *p*-values corrected for multiple comparisons using the Benjamini–Hochberg false-discovery rate (FDR) approach.

**Table 9:** The list and main information of the analyzed SNPs.

SNP ID	SNP Position	Frequency (1000 Genome Project, European)	Assay ID	Context Sequence [VIC/FAM]
rs3847987	chr12:47844285	C = 0.8539 A = 0.1461	C___2404006_20	AAGGGGGTGGGGTGGGAGCTGTGGG[C/A]CGATTATTTATCGTGAGTAGGCAGG
rs731236	chr12:47844974	A = 0.6004 G = 0.3996	C___2404008_10	TGGACAGGCGGTCCTGGATGGCCTC[A/G]ATCAGCGCGGCGTCCTGCACCCCAG
rs7975232	chr12:47845054	C = 0.4453 A = 0.5547	C__28977635_10	AAGGCACAGGAGCTCTCAGCTGGGC[A/C]CCTCACTGCTCAATCCCACCACCCC
rs1544410	chr12:47846052	C = 0.5964 T = 0.4036	C___8716062_20	GAGCAGAGCCTGAGTATTGGGAATG[T/C]GCAGGCCTGTCTGTGGCCCCAGGAA
rs2228570	chr12:47879112	A = 0.3777 G = 0.6223	C__12060045_20	GGAAGTGCTGGCCGCCATTGCCTCC[A/G]TCCCTGTAAGAACAGCAAGCAGGCC
rs11168293	chr12:47899933	G = 0.6789 T = 0.3211	C__44841112_10	AGAGGAGGCTAGAGTCCATTTCTCC[G/T]TCCAGCTGGGCTAGGTTCAGGAGCC

## Data Availability

The data presented in this study are available on request from the corresponding author.

## Supplementary Materials

The following supporting information can be downloaded at: https://www.mdpi.com/article/10.3390/ijms27104217/s1.
