# Supplementary material for: Association of Vitamin D Receptor Gene Polymorphisms with Serum 25-Hydroxyvitamin D Levels in Lithuanian Adults with Atopic Dermatitis: A Case—Control Study
Source: Int J Mol Sci. 2026 May 9;27(10):4217. doi: 10.3390/ijms27104217 (PMC13207046; doi:10.3390/ijms27104217)
Supplement: Supplementary file 1 [file ijms-27-04217-s001.zip › Supplementary Files/Consent Form for genetics (Lithuanian).pdf]

PATVIRTINTA
Lietuvos bioetikos komiteto
biomedicininių tyrimų ekspertų grupės
2016 m. lapkričio 15 d. sprendimu
PAKEISTA
Lietuvos bioetikos komiteto
biomedicininių tyrimų ekspertų grupės
2018 m. sausio 16 d. sprendimu

INFORMUOTO ASMENS SUTIKIMO FORMA

Dėl sutikimo dalyvauti biobanko veikloje

Biomedicininio tyrimo pavadinimas:

Vitamino D ir jo receptorių genų polimorfizmų palyginamasis tyrimas tarp Lietuvos, Latvijos ir Taivano vaikų ir suaugusiųjų, sergančių atopiniu dermatitu ir astma

Protokolo Nr.: LLTADA

Užsakovas: Lietuvos sveikatos mokslų universitetas

Adresas: A. Mickevičiaus g. 9, Kaunas; Tel. +370 37 327201; El. paštas: rektoratas@lsmuni.lt

Užsakovo atstovas: prof. Vaiva Lesauskaitė

Atsakingas tyrėjas[1]: prof. Brigita Šitkauskienė

Tyrimo centro pavadinimas: Lietuvos sveikatos mokslų universiteto ligoninė Kauno klinikos

Adresas: Eivenių g. 2, Kaunas; Tel +370 32 63 75; El. paštas: rastine@kaunoklinikos.lt

1. Kokia šio dokumento paskirtis?

Šioje formoje pateikiama Jums skirta informacija apie dalyvavimą biobanko veikloje.

2. Kodėl atliekami biomedicininiai tyrimai?

Svarbu suprasti, kad nors biomedicininio tyrimo metu Jums bus atliekami sveikatos patikrinimai ir medicininės procedūros, biomedicininis tyrimas iš esmės skiriasi nuo įprastos

[1] Jeigu tyrėjo adresas nesutampa su tyrimo centro adresu – nurodykite abu

(kasdienės) klinikinės praktikos. Įprastos (kasdienės) klinikinės praktikos tikslas yra Jus (t. y. konkretų asmenį, pacientą) išgydyti ir/ar pagerinti Jūsų sveikatos būklę. Pagrindinis biomedicininio (mokslinio) tyrimo tikslas – gauti naujų medicinos mokslo žinių, kurios ateityje padėtų kitų šia liga sergančių pacientų sveikatai. Kitaip tariant, pagrindinis šio tyrimo tikslas nėra tiesioginė nauda Jūsų sveikatai.

3. Kodėl atliekamas šis tyrimas?

Astma ir atopiniu dermatitu serga beveik 300 milijonų žmonių visame pasaulyje. Šių ligų skaičius nuolat auga, todėl mokslininkai stengiasi išsiaiškinti to priežastis. Paskutiniu metu vis daugiau dėmesio skiriama aplinkos ir gyvensenos įtakai įvairių ligų, taip pat ir astmos bei atopinio dermatito, išsivystymui. Pateikiama vis daugiau duomenų apie teigiamą vitamino D reikšmę įvairioms organizmo sistemoms, įskaitant ir imuniniam atsakui. Vitaminas D veikia per vitamino D receptorius, kurie yra išsidėstę daugelyje mūsų organizmo ląstelių, taip pat virškinamojo trakto, kvėpavimo takų ir imuninės sistemos. Keliama hipotezė, kad vitamino D receptorių genetiniai variantai gali būti potencialūs alerginių ligų išsivystymo veiksniai.

Daugėja įrodymų, kad mikroorganizmai taip pat yra svarbūs daugelio ligų patogenezėje. Manoma, kad jie gali turėti įtakos ir imuniniam atsakui. Kai kurie mokslininkai teigia, kad nepakankamas kontaktas su aplinkoje esančiais mikroorganizmais yra viena iš alergijos išsivystymo priežasčių. Ankstyva mikroorganizmų kolonizacija prasideda gimdymo metu. Vėliau mikrofloros formavimasis priklauso nuo higienos, aplinkos veiksnių, mitybos. Vienas veiksnių gali būti ir vitamino D bei vitamino D receptorių kiekis. Be to, vienas iš vitamino D receptorių genų ekspresiją reguliuojančių veiksnių yra žarnyno mikroorganizmų metabolitai.

Šis tarptautinis tyrimas lygins skirtingose geografinėse vietovėse gyvenančias populiacijas, kurioms būdingas skirtingas gyvenimo būdas bei mityba, siekiant įgyti naujų žinių apie vitamino D ir žarnyno mikrobiotos reikšmę astmai ir atopiniam dermatitui, kurios galimai bus svarbios ateityje kuriant naujus šių ligų profilaktikos, prognozės ir gydymo būdus.

4. Kokie asmenys pasirenkami dalyvauti šiame tyrime?

Kviečiame Jus dalyvauti klinikiniame tyrime, nes sergate lengvos ar vidutinės eigos atopiniu dermatitu ir/ ar astma arba esate sveikas asmuo, sutikęs dalyvauti kontrolinėje tiriamųjų grupėje bei atitinkate pagrindinius išvardytus įtraukimo į tyrimą kriterijus. Pagrindiniai įtraukimo į šį tyrimą kriterijai yra šie:

18-60 m. amžius.

Sisteminio poveikio imunosupresinių vaistų (slopinančių imuninę sistemą) nevartojimas bent 1 mėnesį iki tyrimo pradžios.

Sisteminio poveikio antihistamininių vaistų nevartojimas bent 1 savaitę iki tyrimo pradžios.

Nėra onkologinės, autoimuninės ligos, lėtinės ar ūminės infekcijos.

Jūsų dalyvavimas yra svarbus alerginėmis ligomis sergančių asmenų diagnostikos, prognozavimo ir gydymo pagerinimui.

5. Kas atlieka/užsako šį biomedicininį tyrimą?

Šio biomedicininio tyrimo užsakovas yra Lietuvos sveikatos mokslų universitetas. Tyrimas bus atliekamas Lietuvos sveikatos mokslų universiteto ligoninėje Kauno klinikose. Šiam tyrimui atlikti gautos lėšos iš Lietuvos mokslo tarybos pagal bendrą Lietuvos–Latvijos–Kinijos (Taivanas) mokslinių tyrimų programą (sutarties Nr. S-LLT-20-1).

6. Tikimybė patekti į skirtingas tiriamųjų grupes ir dalyvavimo šiose grupėse ypatybės.

Šiame tyrime pacientai bus skirstomi į grupes tik pagal ligos diagnozę (astma sergančių asmenų grupė, atopiniu dermatitu sergantys asmenys ir kontrolinė sveikų tiriamųjų grupė).

7. Kiek truks Jūsų dalyvavimas šiame tyrime?

Bendra tyrimo trukmė – ketveri metai. Jūs į tyrimo centrą turėsite atvykti vieną arba du kartus. Pirmasis vizitas truks 1 valandą, antrasis – 30 minučių.

8. Kokiose šalyse bus vykdomas šis tyrimas?

Lietuvoje, Latvijoje, Taivane.

9. Kiek tiriamųjų dalyvaus numatyta šiame tyrime?

Į tyrimą planuojama įtraukti 170 žmonių – 60 astma sergančių asmenų, 60 atopiniu dermatitu sergančių asmenų ir 50 sveikų asmenų.

10. Ką Jums reikės daryti?

Pirmojo vizito metu bus paimtas periferinis kraujas (20 ml) vitamino D kiekio, vitamino D receptorių polimorfizmų, alergenui specifinių imunoglobulino E kiekio, uždegiminių žymenų ištyrimui. Daliai tiriamųjų (atsitiktine tvarka, tiems, kurie sutiks) bus paimtas išmatų mėginys mikrobiotos tyrimams. Šis tyrimas bus paimtas antrojo vizito metu.

Pagal įprastą klinikinę praktiką Jums būtų paimtas mažesnis kiekis kraujo ir netiriamas vitamino D receptorių genetinis polimorfizmas, žarnyno mikrofloros tyrimas, ne visais atvejais būtų atliekamas vitamino D kiekio kraujyje tyrimas.

11. Ar dalyvavimas biomediciniame tyrime Jums bus naudingas? / Kokios naudos galite tikėtis dalyvaudami šiame tyrime?

Šio tyrimo metu Jūs būsite tiriamas dėl atopinio dermatito ir astmos, bus įvertinta plaučių funkcija, įsijautrinimas alergenams, vitamino D kiekis, jo receptorių genų polimorfizmai, žarnyno mikroflora. Tačiau pagrindinis šio tyrimo tikslas – gauti naujų medicinos mokslo žinių,

kurios ateityje padėtų pacientų, sergančių alergine astma ir/ ar alerginiu rinitu, sveikatai. Kitaip tariant, pagrindinis šio tyrimo tikslas nėra tiesioginė nauda Jūsų sveikatai.

12. Kokia su dalyvavimu šiame tyrime susijusi rizika ir nepatogumai?

Informacija pateikta pagrindinėje informuoto asmens sutikimo formoje.

13. Jei atsitiktų kas nors negero? (Informacija apie draudimą)

Jūs turite teisę į žalos sveikatai ir su tuo susijusios neturtinės žalos, patirtos dalyvaujant šiame tyrime, atlyginimą. Sveikatos priežiūros įstaiga yra sudariusi draudimo sutartį, kurioje numatytas žalos, galinčios atsirasti šio biomedicininio tyrimo metu, atlyginimas.

Su draudimo taisyklėmis galite susipažinti tyrimo vietoje, kreipdamiesi į gydytoją tyrėją. Jei manote, kad tyrimo metu patyrėte žalą, taip pat kreipkitės į gydytoją tyrėją.

14. Ar galėsite nutraukti dalyvavimą tyrime?

Jei nuspręsite pasitraukti iš tyrimo šiam nepasibaigus, tyrėjas pateiks ir paprašys parašyti laisvos formos atsisakymo prašymą.

Jūs turite teisę atsisakyti dalyvauti tyrime, nenurodant priežasčių ir motyvų.

Norėtume atkreipti dėmesį, kad šio tyrimo rezultatai, t. y. tyrimo dokumentuose iki Jūsų sutikimo dalyvauti biomedicininiame tyrime atšaukimo įrašyti duomenys nebus sunaikinti, jei Jūs sutiksite. Priešingu atveju, duomenys bus sunaikinti.

Jeigu dėl pablogėjusios sveikatos būklės negalėsite spręsti apie tolesnes galimybes dalyvauti tyrime, į Jūsų norą atšaukti sutikimą dalyvauti tyrime bus atsižvelgta, bet teisiškai šį sprendimą priims sutuoktinis, jeigu jo nėra – vienas iš tėvų, pilnamečių vaikų arba kitas teisėtas (Jūsų) atstovas.

15. Jūsų dalyvavimo tyrime nutraukimo aplinkybės ir kriterijai

Jei nesilaikysite tyrėjo nurodymų ar dalyvaujant tyrime pablogės Jūsų sveikatos būklė, Jūs daugiau nebegalėsite dalyvauti tyrime.

16. Kokias pasirinkimo galimybes turėsite, jeigu nesutiksite dalyvauti šiame tyrime arba atšauksite sutikimą jame dalyvauti?

Tyrime dalyvaujate savanoriškai, todėl turite teisę atsisakyti, o pradėjęs galite bet kada iš jo pasitraukti.

Jūsų sprendimas atsisakyti dalyvauti ar nutraukti dalyvavimą tyrime nedarys jokios įtakos teikiamai įprastinei sveikatos priežiūrai.

Visiems tiriamiesiems bus skiriama įprastinė sveikatos priežiūra, nepriklausomai nuo to, ar jie dalyvaus šiame tyrime, ar ne.

Informacija apie asmens duomenų konfidencialumą, kas ir kokiu tikslu galės susipažinti su Jūsų asmens duomenimis, kiek laiko bus saugomi tyrimo metu surinkti duomenys ir kas už tai bus atsakingas aprašyti pagrindinėje informuoto asmens sutikimo formoje.

21. Kas įvertino šį biomedicininį tyrimą? Į ką kreiptis, jeigu iškiltų klausimų?

Dėl savo kaip tyrimo dalyvio teisių galite kreiptis į leidimą atlikti šį biomedicininį tyrimą išdavusį Kauno regioninį biomedicininių tyrimų etikos komitetą, Lietuvos sveikatos mokslų universitetas, Mickevičiaus g. 9, LT-44307, Kaunas, tel. (8-37) 326889, el. paštas: kaunorbtek@lsmuni.lt.

Dėl informacijos apie duomenų tvarkymą galite kreiptis į Valstybinę duomenų apsaugos inspekciją, A. Juozapavičiaus g. 6, LT-09310 Vilnius, tel. (8-5) 2127535, el. paštas: ada@ada.lt.>

SUTIKIMAS DALYVAUTI BIOMEDICININIAME TYRIME

Aš perskaičiau šią Informuoto asmens sutikimo formą ir supratau man pateiktą informaciją.
Man buvo suteikta galimybė užduoti klausimus ir gavau mane tenkinančius atsakymus.
Supratau, kad galiu bet kada pasitraukti iš tyrimo, nenurodydama(s) priežasčių.
Supratau, kad norėdama(s) atšaukti sutikimą dalyvauti biomediciniame tyrime, raštu turiu apie tai informuoti tyrėją/kitą jo įgaliotą biomedicininį tyrimą atliekantį asmenį.
Patvirtinu, kad turėjau užtektinai laiko apsvarstyti man suteiktą informaciją apie biomedicininį tyrimą.
Supratau, kad dalyvavimas šiame tyrime yra savanoriškas.
Patvirtinu, kad sutikimą dalyvauti šiame biomediciniame tyrime duodu laisva valia.
Leidžiu naudoti asmens duomenis ta apimtimi ir būdu, kaip nurodyta Informuoto asmens sutikimo formoje.
Patvirtinu, kad gavau Informuoto asmens sutikimo formos egzempliorių, pasirašytą tyrėjo/ kito jo įgalioto biomedicininį tyrimą atliekančio asmens.

Asmuo (ar kitas sutikimą turintis teisę duoti asmuo)

______________	______________	______________	__________	______________	_:_
vardas	pavardė	atstovavimo pagrindas	parašas	pasirašymo data	pasirašymo laikas

Patvirtinu, kad suteikiau informaciją apie biomedicininį tyrimą aukščiau nurodytam asmeniui.
Patvirtinu, kad asmeniui (ar kitam sutikimą duoti turinčiam teisę asmeniui) buvo skirta pakankamai laiko apsispręsti dalyvauti biomediciniame tyrime, atsižvelgiant į biomedicininio tyrimo pobūdį, taip pat įvertinus kitas aplinkybes, galinčias daryti įtaką priimamam sprendimui.
Aš skatinau asmenį (ar kitą sutikimą turintį teisę duoti asmenį) užduoti klausimus ir į juos atsakiau.

Tyrėjas ar kitas jo įgaliotą biomedicininį tyrimą atliekantis asmuo

______________	______________	______________	__________	______________	_:_
vardas	pavardė	pareigos tyrime	parašas	pasirašymo data	pasirašymo laikas
