# Supplementary material for: Association of Vitamin D Receptor Gene Polymorphisms with Serum 25-Hydroxyvitamin D Levels in Lithuanian Adults with Atopic Dermatitis: A Case—Control Study
Source: Int J Mol Sci. 2026 May 9;27(10):4217. doi: 10.3390/ijms27104217 (PMC13207046; doi:10.3390/ijms27104217)
Supplement: Supplementary file 1 [file ijms-27-04217-s001.zip › Supplementary Files/General Consent Form - English.pdf]

VERIFIED
Lithuanian Bioethics Committee
Biomedical Research Expert Groups
By decision of 15 November 2016
CHANGED
Lithuanian Bioethics Committee
Biomedical Research Expert Groups
By decision of 16 January 2018

INFORMED CONSENT FORM

Name of the biomedical study:

Comparative study of vitamin D and its receptor gene polymorphisms among children and adults with atopic dermatitis and asthma from Lithuania, Latvia and Taiwan

Protocol No: LLTADA

Client: Lithuanian University of Health Sciences

Address: A. Mickevičiaus g. 9, Kaunas; Phone: +370 37 327201; E-mail: rektoratas@lsmuni.lt

Client's representative: prof. Vaiva Lesauskaitė

Principal investigator[1]: prof. Brigita Šitkauskienė

Name of the research centre: Kaunas Clinics Hospital of the Lithuanian University of Health Sciences

Address: Eivenių g. 2, Kaunas; Phone +370 32 63 75; E-mail: rastine@kaunoklinikos.lt

1. What is the purpose of this document?

This form provides you with information about the biomedical research, discusses the reasons for conducting the research, scientific research procedures, benefits, risks, possible inconveniences and other important information. If you choose to participate, we will ask you

[1] If the investigator's address does not match the address of the study centre, please indicate both

to sign this consent form, which means you agree to follow the instructions of the medical examiner and the research team during the study. By signing this document, you agree to participate in the research. Take your time and read this document carefully, if you do not understand any word or statement, be sure to ask any questions you may have to the examination doctor or other members of the research team. You can consult with family, friends, or your doctor before making a decision.

2. Why are biomedical/clinical studies being conducted?

It is important to understand that although you will undergo health checks and medical procedures during the biomedical examination, the biomedical examination is fundamentally different from normal (daily) clinical practice. The goal of routine (daily) clinical practice is to cure you (i.e. a specific person, patient) and/or improve your state of health. The main goal of biomedical (scientific) research is to obtain new knowledge of medical science that would help the health of other patients with this disease in the future. In other words, the main goal of this study is not the direct benefit to your health.

3.Why is this test performed?

Asthma and atopic dermatitis affect almost 300 million people worldwide. The number of these diseases is constantly growing, so scientists are trying to find out the reasons for this. Recently, more and more attention has been paid to the influence of the environment and lifestyle on the development of various diseases, including asthma and atopic dermatitis. There is more and more data on the positive importance of vitamin D for various body systems, including the immune response. Vitamin D works through vitamin D receptors, which are located in many cells of our body, as well as the gastrointestinal tract, respiratory tract, and immune system. It is hypothesized that genetic variants of vitamin D receptors may be potential factors in the development of allergic diseases.

There is growing evidence that microorganisms are also important in the pathogenesis of many diseases. It is believed that they can also affect the immune response. Some scientists argue that insufficient contact with microorganisms in the environment is one of the reasons for the development of allergies. Early colonization of microorganisms begins during childbirth. Subsequently, the formation of microflora depends on hygiene, environmental factors, nutrition. One of the factors may be the amount of vitamin D and vitamin D receptors. In addition, one of the factors that regulate the gene expression of vitamin D receptors is metabolites of intestinal microorganisms.

This international study will compare populations living in different geographical areas with different lifestyles and diets in order to gain new knowledge about the role of vitamin D and the intestinal microbiota in asthma and atopic dermatitis, which may be important in the future development of new methods of prevention, prognosis and treatment of these diseases.

4. Which individuals are selected to participate in this study?

We invite you to participate in a clinical trial because you have mild to moderate atopic dermatitis and/or asthma or you are a healthy person who has agreed to participate in a control group of subjects and meets the main criteria listed for enrollment in the study. The main criteria for inclusion in this study are:

Age 18-60.

Absence of systemic immunosuppressive drugs (immunosuppressants) for at least 1 month prior to the start of the study.

Absence of systemic antihistamines for at least 1 week prior to the start of the study.

There is no oncological, autoimmune disease, chronic or acute infection.

Your participation is important for improving the diagnosis, prognosis and treatment of people with allergic diseases.

5. Who performs/orders this biomedical test?

The commissioner of this biomedical study is the Lithuanian University of Health Sciences. The study will be carried out at the Lithuanian University of Health Sciences Hospital Kaunas Clinics. Funds for this research were received from the Research Council of Lithuania under the joint Lithuanian-Latvian-Chinese (Taiwan) research programme (contract No. S-LLT-20-1).

6. Probability of entering different groups of subjects and characteristics of participation in these groups.

In this study, patients will be grouped only according to the diagnosis of the disease (asthma group, atopic dermatitis and healthy control group).

7. How long will your participation in this study last?

The total duration of the study is four years. You will need to come to the test centre once or twice. The first visit will last 1 hour, the second – 30 minutes.

8. In which countries will this study be conducted?

Lithuania, Latvia and Taiwan.

9. How many subjects will participate in this study?

It is planned to include 170 people in the study – 60 asthmatics, 60 people with atopic dermatitis and 50 healthy people.

10. What will you need to do?

During the first visit, the inclusion criteria will be assessed. Patients with atopic dermatitis will be evaluated with the SCORAD index, asthma subjects will be evaluated by an asthma control test and a standardised asthma quality of life questionnaire (AQLQ(S)). All subjects will undergo a physical examination, skin prick samples with standard allergens (to assess sensitization), spirogram (to assess lung function), peripheral blood collection (20 ml) (for testing for vitamin D levels, vitamin D receptor polymorphisms, allergen-specific immunoglobulin E levels, inflammatory markers). A part of the subjects (randomly, those who agree) will be given a stool sample for microbiota testing. This study will be taken during the second visit.

According to normal clinical practice, you should come to the test centre once or twice, you would have a smaller amount of blood taken and the genetic polymorphism of vitamin D receptors, the intestinal microflora test, and not all cases would have a blood vitamin D test performed.

11. Will participating in a biomedical study benefit you? / What benefits can you expect from participating in this study?

During this test, you will be tested for atopic dermatitis and asthma, lung function, sensitization to allergens, vitamin D levels, gene polymorphisms of its receptors, intestinal microflora will be assessed. However, the main goal of this study is to obtain new medical science knowledge that would help the health of patients with allergic asthma and/or allergic rhinitis in the future. In other words, the main goal of this study is not the direct benefit to your health.

12. What are the risks and inconveniences associated with participating in this study?

Possible risks and inconveniences:

Short-term discomfort or other unpleasant sensations associated with the following procedures:

- assessment of lung function by performing a spirogram (the subject may feel short-term shortness of breath, dizziness due to deep breathing);
- skin puncture samples (the subject may feel short-term slight pain, itching);
- peripheral blood collection during a peripheral vein puncture (the subject may feel temporary pain or unpleasant sensation, in rare cases, a hematoma may form at the puncture site, which disappears within a few weeks);

If, due to unforeseen circumstances (force majeure or force majeure, criminal acts of third parties, etc.) that are not known to the investigator and over which the investigator cannot

influence, confidential information becomes available to third parties to whom you have not given your consent, the investigator will immediately inform you about it. However, the researcher will make every effort to ensure that your personal data processed for the purpose of this biomedical research is not accessible to third parties to whom you have not given your consent and will implement data security measures to protect personal data from accidental or unlawful disclosure, as well as from any other unlawful processing.

13. What if something bad happens? (Insurance information)

You are entitled to compensation for damage to health and related non-material damage suffered as a result of participating in this investigation.

The health care institution has concluded an insurance contract that provides for compensation for damages that may occur during this biomedical study.

You can get acquainted with the insurance rules at the examination site by contacting an investigator doctor. If you think you have suffered harm during the examination, you should also consult an investigator doctor.

14. Will you be able to stop participating in the study?

If you decide to withdraw from the investigation before it is over, the investigator will submit and ask you to write a free-form waiver request.

You have the right to refuse to participate in the survey without giving reasons or reasons.

We would like to point out that the results of this study, i.e. the data recorded in the study documents prior to the cancellation of your consent to participate in the biomedical study, will not be destroyed if you agree. Otherwise, the data will be destroyed.

If you are unable to decide on further opportunities to participate in the study due to a deteriorated state of health, your wish to withdraw your consent to participate in the study will be taken into account, but legally this decision will be made by your spouse, if he or she is not present – one of the parents, adult children or another legal representative (your).

15. Circumstances and criteria for terminating your participation in the investigation

If you do not follow the investigator's instructions or if your health deteriorates while participating in the test, you will no longer be able to participate in the test.

16. What choices will you have if you do not agree to participate in this study or withdraw your consent to participate in it?

You participate in the study voluntarily, so you have the right to opt out, and you can opt out at any time once you start.

Your decision to refuse or stop participating in the study will not affect the routine healthcare provided.

All subjects will receive routine health care, regardless of whether they participate in this study or not.

17. Will you incur any costs as a result of participating in this study?

There is no fee for participation in biomedical research. Compensation for expenses incurred or time spent is not provided for in this study.

18. Will your personal data be confidential?

The health information obtained during the biomedical research, which allows to establish the identity of a person, is confidential and may be provided only in accordance with the procedure established by the laws regulating the rights of patients and the protection of personal data.

The Data Controller is the Lithuanian University of Health Sciences Hospital Kauno Klinikos, company code 135163499, address – Eivenių str. 2, Kaunas.

In order to protect the confidentiality of your data, you will be provided with a special code, which will be indicated in all documents except for the consent form.

Computers that store electronic test documents and data are password protected. Login codes are known only to researchers, this data is updated monthly.

The documents are stored in a lockable closet, the key to which only the investigators have.

19. Who will be able to access your personal data and for what purpose?

By signing this form, you agree that research center researchers, research control bodies (such as ethics committees), and authorized research supervisors of the study sponsor will have access to all information collected about you for the purposes of this study.

The data collected will only be used by the trial physicians for the purposes of this clinical trial. The aggregated data will be published in scientific articles and reports without the possibility of revealing the identity of the subjects.

You have the right to know what data has been collected, and you can also request the rectification, destruction or suspension of the processing of your personal data if you decide to withdraw from the investigation at an earlier time.

20. How long will the data collected during the investigation be stored and who will be responsible for it?

All information will be recorded in electronic and paper documents drawn up specifically for the clinical trial and stored in the study centre for 5 years after the end of the study. This is the time to store the data in accordance with the procedure established by the customer in order

to ensure the quality and control of the data. Later, your personal data will be destroyed in accordance with the procedure established by the investigation center. The principal investigator will be responsible for storing documents at the research centre.

21. Who evaluated this biomedical study? Who should I contact if I have any questions?

For your rights as a participant in the study, you can apply to the Kaunas Regional Biomedical Research Ethics Committee, Lithuanian University of Health Sciences, Mickevičiaus str. 9, LT-44307, Kaunas, tel. (8-37) 326889, e-mail: kaunorbtek@lsmuni.lt.

For information on data processing, you can contact the State Data Protection Inspectorate, A. Juozapavičiaus str. 6, LT-09310 Vilnius, tel. (8-5) 2127535, e-mail: ada@ada.lt.>

CONSENT TO PARTICIPATE IN BIOMEDICAL RESEARCH

I have read this Informed Consent Form and understood the information provided to me.

I was given the opportunity to ask questions and received answers that satisfied me.

I realized that I could withdraw from the investigation at any time without giving reasons.

I understood that in order to withdraw my consent to participate in a biomedical study, I had to inform the researcher/other biomedical researcher authorised by him or her in writing.

I confirm that I have had sufficient time to consider the information provided to me about the biomedical study.

I realized that participation in this study is voluntary.

I confirm that I give my consent to participate in this biomedical research of my own free will.

I authorise the use of personal data to the extent and in the manner specified in the Informed Consent Form.

I confirm that I have received a copy of the Informed Person Consent Form signed by the researcher/other biomedical researcher authorised by him/her.

Person (or other person entitled to give consent)

					_:_
Name	Surname	Basis for representation	Signatur e	Date of signature	Signing time

I confirm that I have provided information about the biomedical research to the person mentioned above.

I confirm that the person (or other person entitled to give consent) has been given sufficient time to decide to participate in the biomedical research, taking into account the nature of the biomedical research, as well as after assessing other circumstances that may influence the decision taken.

I encouraged the person (or other person with the right to give consent) to ask questions and answered them.

Researcher or other person conducting biomedical research authorized by him or her

					_:_
Name	Surname	Responsibilitie s Research	Signature	Date of signature	Signing time
